# A Sexual Dimorphism in the Spatial Vision of North American Band-Winged Grasshoppers

**DOI:** 10.1093/iob/obab008

**Published:** 2021-04-07

**Authors:** A B Duncan, B A Salazar, S R Garcia, N C Brandley

**Affiliations:** 1 Department of Organismal Biology and Ecology, Colorado College, 14 E, W Cache La Poudre Street, Colorado Springs, CO 80903, USA; 2 Department of Biology, College of Wooster, 1189 Beall Avenue, Wooster, OH 44691, USA

## Abstract

Visual acuity (VA)—a measurement of the fineness or coarseness of vision—may vary within a species including between the biological sexes. Although numerous studies have found males with finer VA than females, relatively few have shown the opposite with females having finer vision. This is surprising because our understanding of between species differences in VA suggests that females may have finer vision than males if they 1) are larger than males, or 2) need finer vision to detect and/or discriminate between males. Here, we estimate the interommatidial angle (ΔΦ, an anatomical measurement of VA) in three species of band-winged grasshoppers in which females are both the larger sex and likely interpret visual signals (*Arphia pseudonietana*, *Dissosteira carolina*, and *Spharagemon equale*; total *n* = 98). Using a radius of curvature estimation method, we find that females have ∼19% finer estimated ΔΦ than males in the most acute region and axis of the eye, but that this dimorphism varies between species. Further visual explorations of the species showing the greatest body size dimorphism (*D. carolina*) suggest that this ΔΦ dimorphism is driven by females having larger eyes with more ommatidia. In contrast to many diurnal flying insects where males have finer vision to acquire mates, our study is one of the first to demonstrate a female-biased sexual dimorphism in acuity. Given 1) the number of species in which females are larger than males, and 2) the variability of mating behaviors across taxa, our results suggest that differences in VA between the sexes may be more common than currently appreciated.

**Resumen** La agudeza visual (AV)—una medida de la finura o la dificultad visual—puede variar dentro de una especie, incluso entre los sexos biológicos. Aunque numerosos estudios han encontrado machos con una AV más fina que las hembras, relativamente pocos han demostrado lo contrario, hembras con visión más fina. Esto es sorprendente porque nuestra comprensión de diferencias entre especies en AV sugiere que las hembras pueden tener una visión más fina que los machos si 1) son más grandes que los machos, o 2) necesitan una visión más fina para detectar y/o discriminar entre los machos. Aquí, estimamos el ángulo interommatidial (Δ*Φ*, una medida anatómica de AV) en tres especies de saltamontes de ala de banda en las que las hembras son el sexo más grande y probablemente interpretan señales visuales (*Arphia pseudonietana*, *Dissosteira carolina*, y *Spharagemon equale*; total *n* = 98). Usando un método de estimación de radio de curvatura, encontramos que las hembras tienen un estimado Δ*Φ* ∼19% más fino que los machos en la región y eje más agudos del ojo, pero que este dimorfismo varía entre especies. Exploraciones visuales adicionales de la especie que muestra el mayor dimorfismo del tamaño corporal (*D. carolina*) sugieren que este dimorfismo de ΔΦ debe a que las hembras tienen ojos más grandes con más omatidios. En contraste con muchos insectos voladores diurnos donde los machos tienen una visión más fina para adquirir parejas, nuestro estudio es uno de los primeros en demostrar un dimorfismo sesgado por las hembras en la agudeza. Dado 1) el número de especies en las que las hembras son más grandes que los machos, y 2) la variabilidad de los comportamientos de apareamiento entre taxones, nuestros resultados sugieren que las diferencies de AV entre los sexos pueden ser más comunes de lo que se aprecia actualmente.

## Introduction

An animal’s behavior is driven by what information they can perceive, which itself is limited by their sensory systems (Partan and Marler 2002; von Uexküll 2013). Therefore, studying how an animal responds to stimuli requires understanding what information it can perceive (Jordan and Ryan 2015; Caves et al. 2019). Notably, animals differ not just in the presence or absence of senses, but in the fineness with which they can parse information within a sensory modality. For example, visual acuity (VA)—defined as the ability to perceive static spatial detail and used as a measurement of the fineness or coarseness of vision—varies by up to four orders of magnitude between species (Land and Nilsson 2012; Caves et al. 2018), making it a promising parameter for further exploration of within sense variation.

VA varies not only between species, but also varies within a species, including between the sexes. For example, numerous studies of insects have suggested that males may have finer VA than females in specialized eye regions designed to spot potential mates (Zeil 1983; Land and Eckert 1985; Merry et al. 2006; Lau and Meyer-Rochow 2007; Rutowski et al. 2009). In contrast, published examples of females having finer VA than males are rare, with current examples in insects either showing small overall differences (Bergman and Rutowski 2016) or being limited to miniaturized eyes with very coarse vision regardless of sex (Fischer et al. 2011). This lack of female-biased VA is especially surprising considering how VA changes between species, with both size differences and behavioral needs having the potential to lead to female-biased VA.

Despite the lack of published examples of female-biased VA, our understanding of between species differences in VA shows multiple scenarios that could lead to female-biased VA. The first scenario relies on the relationship between size and VA. Because of the physical limits of the eye (Barlow 1952; Kirschfeld 1976; Land 1997), larger eyes (and through correlations between eye and body size, larger bodies) are typically associated with finer vision regardless of taxonomic level, with studies supporting this at the kingdom (Land and Nilsson 2012; Caves et al. 2018), superclass/class (Kiltie 2000; Veilleux and Kirk 2014; Caves et al. 2017), order (Rutowski et al. 2009), and family (Jander and Jander 2002) levels. Although fewer studies have examined whether the same principle holds within a species (Spaethe and Chittka 2003; Corral-López et al. 2017; Taylor et al. 2019), this suggests that females could have finer vision than males when they are the larger sex. Another scenario relies on behavioral differences. Behaviorally, VA often changes with the needs of an animal such as being finer in predators (Veilleux and Kirk 2014), in animals in high-photon environments (Veilleux and Kirk 2014; Caves et al. 2017), or as a result of sexual selection (Kirschfeld and Wenk 1976; Land and Eckert 1985). Notably, mating behaviors vary between animals, and situations where females need to locate or discriminate among mates could lead to them having finer vision than males.

The North American band-winged grasshoppers are an ideal animal group for the exploration of female-biased VA because 1) females are often larger than males and 2) their mating behaviors (although variable) often involve female discrimination of fine visual signals (Otte 1970, 1984). Band-winged grasshoppers (subfamily Oedipodinae) are a morphologically diverse globally distributed subfamily of ∼200 diurnal species known for their colorful hindwing patterns. Previous work on the vision of band-winged grasshoppers has been limited to species found in the Eastern Hemisphere and has not examined differences between the sexes (Burtt and Catton 1954; Horridge 1978; Krapp and Gabbiani 2005).

To determine if females have finer vision than males in North American band-winged grasshoppers, we first use a radius of curvature estimation technique (Bergman and Rutowski 2016) to estimate the interommatidial angle (an anatomical measurement of VA) in the most acute region of the eye across three species [*Arphia pseudonietana* (Thomas, 1870), *Dissosteira carolina* (Linnaeus, 1758), and *Spharagemon equale* (Say, 1825)]. This relatively new method for estimating interommatidial angle allows us to generate a larger sample size than seen in most studies on VA, while relying on the close association between interommatidial angle and acceptance angle seen in many diurnal insects (Land 1997). Then in a second procedure designed to better understand whole eye changes between the sexes, we further examine eye and receptor scaling in the species that showed the greatest size dimorphism (*D. carolina*).

## Materials and methods

### Interommatidial angle variation by biological sex across three species

#### Study organisms

To examine how biological sex (hereafter just sex) influences interommatidial angle (*ΔΦ*) in band-winged grasshoppers, individuals of three species were collected during the summer and early fall (June–October) of 2016 and 2017 in Colorado Springs, CO. *Dissosteira carolina* (*n* = 16 males and 16 females) were collected from an urban site near manicured grass, while *A. pseudonietana* (*n* = 18 males and 18 females) and *S. equale* (*n* = 15 males and 15 females) were collected at a high-altitude grassland site. Grasshoppers were euthanized using ethyl acetate and then stored at approximately −20°C prior to imaging. Total length (head to the tip of the forewings; mm), weight (g), and sex were recorded for each individual.

#### Estimating interommatidial angle (Δ*Φ*) and VA

We measured *ΔΦ* of all three species via a modified radius of curvature estimation (Bergman and Rutowski 2016). Briefly, this method estimates *ΔΦ* by calculating how many ommatidia view a given angle of visual space.

**Fig. 1:**
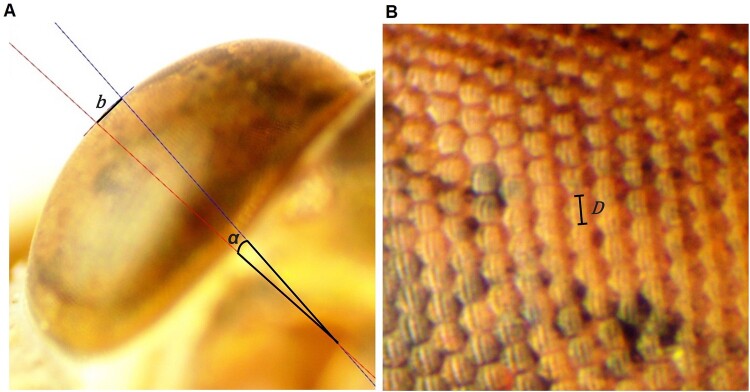
An example of the radius of curvature estimation used to estimate interommatidial angle in band-winged grasshoppers. **A**) A lateral view of the eye with the edge in focus. Small line segments tangent to the curvature of the eye (small red and blue lines at the surface of the eye) have been drawn for the flattest region of the eye. These are used to draw lines perpendicular to the eye surface (longer red and blue lines) which can estimate the curvature of the eye in the relevant axis (see methods and Bergman and Rutowski [2016] for more details). **B**) An anterior view of the flattest region of the eye used to calculate average facet diameter. *b/α* = eye surface length (*b*) in a given angle (*α*), *D* = facet diameter.

To estimate *ΔΦ*, eyes were imaged at 40× magnification using AmScopeX for Mac MU (MW Series 05/26/2016; United Scope LLC, Irvine, CA, USA) under diffuse lighting conditions (LED312DS; Fotodiox Inc., Gurnee, IL, USA) with a M28Z zoom stereo binocular microscope (Swift Optical Instruments Inc., Carlsbad, CA, USA) and AmScope 14MP USB3.0 digital camera (United Scope LLC). Because grasshopper eyes are not spherical and therefore have different curvatures in each axis, using the RCE method requires obtaining images of each curvature separately (Bergman and Rutowski 2016; Bagheri et al. 2020). We used a lateral image to measure curvature—and ultimately *ΔΦ*—in the axis perpendicular to the horizon (*y*-axis), a dorsal view for the axis parallel to the horizon (*x*-axis), and one anterior view of the flattest region of the eye to measure facet diameter (*D*; Fig. 1). For consistent positioning, physical attributes were used for orientation in each image (lateral image = the inside eye edge, dorsal image = the top of the eye, anterior image = the center of the eye).

Using the lateral (for *y* curvature) or dorsal views (for *x* curvature), the localized curvature of the eye (*b*/*α*) was calculated in two axes via ImageJ (v. 1.50i; Schneider et al. 2012). First, we drew two smaller line segments tangent to the surface of the eye with centers roughly 0.2 mm apart (Fig. 1A; Bergman and Rutowski 2016). Longer lines were then drawn that were perpendicular to the original line segments (and thus the eye surface) and bisected the middle of each segment (Fig. 1A). From these lines, *b* was calculated as the distance between the two points created by the intersection of the perpendicular lines to the eye edge, while the angular distance covered between these points (*α*) was calculated using the ImageJ angle function. These measurements were then combined (*b*/*α*) to calculate the distance of the eye’s surface covered in a given angle (µm per °).

Facet diameter (*D*) was calculated in the flattest region of the eye near where curvature was measured (Fig. 1B). Ten facets in each of two axes were measured and then average to compute *D.* Similar to studies in other Oedipodinae (Horridge 1978), preliminary results found that *D* was relatively constant across the majority of the eye surface of each individual grasshopper.

Using the previous measurements, the inter-ommatidial angle (*ΔΦ*) in each axis was then estimated as

(1) ΔΦ=D/(b/α),

where *D* = the facet diameter and *b/α* = eye surface length in a given angle.

Finally, VA (*n* degrees, smaller values indicate finer vision) in each axis was calculated as two times the inter-ommatidial angle (Land 1997; Caves et al. 2018):

(2) VA=2*ΔΦ.

#### Statistical analysis

All data were analyzed in R version 3.4.4 (R CORE Team 2018). To quantify the magnitude of the sexual size dimorphism, we first used a two-way ANOVA examining how total length varies with species and sex.

Estimated *ΔΦ* was analyzed first via a general linear model (GLM) that looked simultaneously at the effects of multiple variables on *ΔΦ*. We used the Akaike information criterion (AIC; Akaike 1974) to rank models, computed the relative likelihood of each model (*l_i_*) compared with all generated models via differences in AIC, and finally computed the probability of each model being the best (*w_i_*) of the set of models examined (Burnham et al. 2011). Next, because our GLM results suggested that sex and species were the main predictors of estimated *ΔΦ*, we used two-way ANOVAs to examine solely how these two factors influence the determinants of *ΔΦ* (such as eye curvature and facet size). Normality for all data were first checked using the Shapiro–Wilk normality test and visual inspection of residuals. To restore normality in three cases, data were either natural log transformed (estimated *ΔΦ_x_*) or had one to two outliers excluded (estimated *ΔΦ_y_*, vertical eye curvature). Student’s *t*-tests or Tukey’s HSDs were used for *post hoc* analysis.

### Eye size and facet count variation in *D. carolina*

#### Study organisms

To further explore the relationship between body size, eye size, and *ΔΦ*, we examined eye and ommatidia scaling within the species that showed the greatest body size dimorphism (*D. carolina*). Unfortunately, logistical issues prevented us from returning to the same study population, so 48 *D. carolina* (*n* = 25 male and 23 female) were collected during the summer (June–September) of 2018 from a suburban site near manicured grass in Wooster, OH. Directly prior to imaging, grasshoppers were euthanized in a freezer for ∼1 h. Weight (g), head to abdomen length (anterior of the head to the posterior tip of the abdomen; mm), head size (mm), and sex were recorded for each individual.

#### Eye size measurements in *D. carolina*

All individuals (25 males and 23 females) were photographed at a lower resolution (30×) than in the previous section to ensure that all eyes completely fit within the images. Eye images were taken using an AmScope stereo trinocular microscope (United Scope LLC) paired with a MU 1403 digital camera (United Scope LLC; MU1403). Because band-winged grasshoppers have non-spherically shaped eyes, calibrated images of each eye were taken from three different angles (anterior, dorsal, and lateral) and used to calculate maximum eye size in all three axes (*x*, *y*, and *z*) in ImageJ (v 1.8.0,  Schneider et al. 2012). Note that this methodology only estimates the exposed eye depth in the *z*-axis.

#### Facet counts in *D. Carolina*

To examine if sexes vary in number of ommatidia, we calculated the total number of facets per eye via eye castings of a subsample of the Ohio *D. carolina* population (e.g., Narendra et al. 2013). Individuals representative of the various eye sizes within each sex were used (*n* = 3 of each sex). A single thin layer of #800 crystal clear nail polish (Sally Hansen Inc., New York, NY, USA) was applied to the entirety of the left eye and dried for 30–50 min. When dry, the eye castings were removed and cut horizontally and vertically into multiple sections with a #11 surgical scalpel blade (Swann-Morton Ltd, Sheffield, UK). Each section was then flattened between two microscope slides overnight before imaging at 30× magnification (Supplementary Fig. S1) using an AmScope stereo trinocular microscope (United Scope LLC) paired with a MU1403 digital camera (United Scope LLC). Each eye section was assigned a non-identifying name and facets were then counted by individuals blinded to original sex and grasshopper ID of the images. Sections originating from the same eye were then summed to determine the total facet/ommatidia count for each eye.

#### Statistical analysis

We examined how sex and eye axis vary via a two-way ANOVA in R (R CORE Team 2018). Student’s *t*-tests or Tukey’s HSDs were used for *post hoc* analysis.

## Results

The data underlying this article are available in the Dryad Digital Repository (doi: 10.5061/dryad.bcc2fqzc3).

### Interommatidial angle variation by sex across three species

#### Dimorphism in body size

**Fig. 2:**
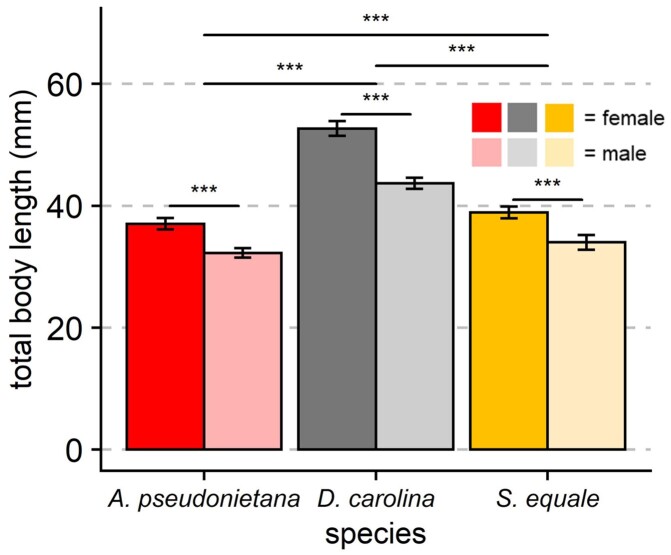
Body length in three species of band-winged grasshoppers. All three species show a sexual size dimorphism, with biological females being longer than males (*P* < 0.001, df = 1, *F* = 257.93, two-way ANOVA). Additionally, total length varies between species, with all three showing significant differences from each other (*P* < 0.001 in all comparisons, Tukey’s HSD). There is also a significant interaction between sex and species on total body length (*P* < 0.001, df = 2, *F* = 12.71). Sample sizes (from left to right) are 18, 18, 16, 16, 15, and 15 individuals, respectively. Error bars indicate 95% CI, significance symbols are based on *post hoc* analyses (Student’s *t*-test for sex differences within a species, Tukey’s HSD for species differences).

Sex, species, and their interaction all significantly predicted total length in band-winged grasshoppers (Fig. 2). Sexes significantly differed in total body length (*P* < 0.001, df = 1, *F* = 257.93, two-way ANOVA), with females being on average 6.2–7.0 mm longer than males (*P* < 0.001, Tukey’s HSD). Additionally, all three species significantly differed from each other in total length (*P* < 0.001, df = 2, *F* = 482.81, two-way ANOVA; all Tukey’s HSD *P* < 0.001). The interaction between sex and species on total body length was also significant (*P* < 0.001, df = 2, *F* = 12.71), with *D. carolina* showing the largest dimorphism.

#### Interommatidial angle perpendicular to the horizon (Δ*Φ_y_*)

**Fig. 3:**
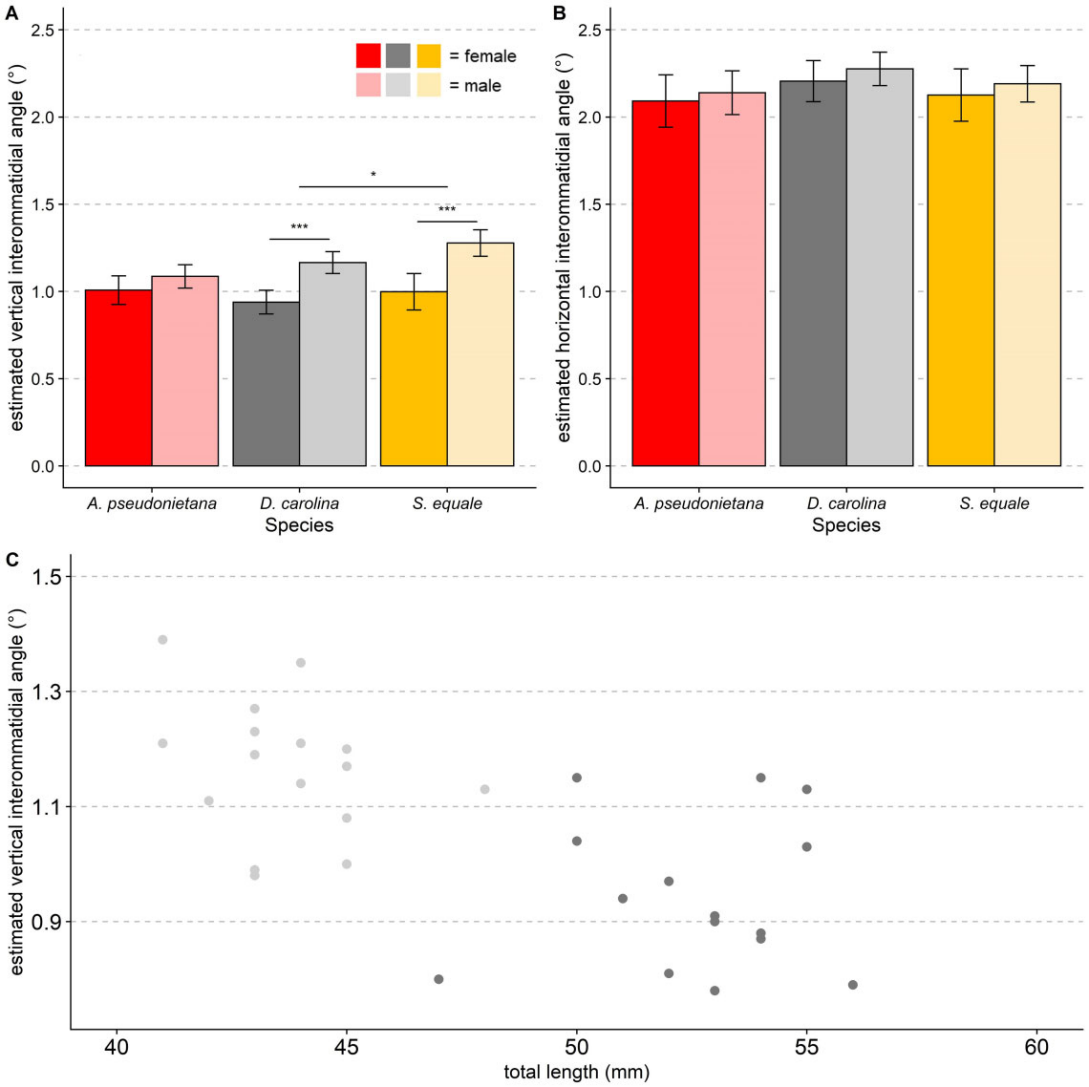
Interommatidial angle (*ΔΦ*) in the two axes of vision in band-winged grasshoppers. *ΔΦ* in band-winged grasshoppers is sexually dimorphic, but only in one axis. Units are degrees, so finer vision is indicated by smaller values. **A**) Biological females typically have finer *ΔΦ* than males in the axis perpendicular to the horizon (*P* < 0.001, df = 1, *F* = 39.97, two-way ANOVA). Additionally, species differ significantly from one another (*P* = 0.034, df = 2, *F* = 3.44) with *A. pseudonietana* having finer vision than *S. equa*le (*P* < 0.049, Tukey’s HSD). Sample sizes (from left to right) are 18, 17, 16, 16, 15, and 14 individuals, respectively. **B**) In contrast to A, *ΔΦ* parallel to the horizon is both coarser and shows no significant sexual dimorphism or species-specific effect (see text for details). Sample sizes (from left to right) are 18, 18, 16, 16, 15, and 15 individuals, respectively. **C**) Although there are negative correlations between total length and *ΔΦ_y_* within a species (here in *D. carolina*, *r*^2^ = 0.44, estimated slope confidence interval of −0.012 to −0.003, *P* < 0.0001, linear regression), this relationship disappears when looking within a sex (all *r*^2^ <0.2, see Supplementary Fig. S2 for all species/sex combos corresponding statistics). **A** and **B**) Error bars indicate 95% CI, significance symbols are based on *post hoc* analyses (Student’s *t*-test for sex differences within a species, Tukey’s HSD for species differences).

Interommatidial angle varied depending on the axis of view in all three species; among all individuals measured (*n* = 98) *ΔΦ* perpendicular to the horizon (*ΔΦ_y_*; average of 1.09°) was approximately half the value of *ΔΦ* parallel to the horizon (*ΔΦ_x_*; average of 2.17°; Fig. 3). As such, results are presented separately for each axis of view.

*ΔΦ_y_* values included two outlier males with especially coarse vision (*ΔΦ_y_* = 1.65° and 1.68°) that were removed from further analysis. Even with these especially coarse males removed, females had finer vision than males. The most parsimonious models of *ΔΦ_y_* all included sex, species, and their interactions as factors (Table 1). Cumulatively, models including both sex and species as predictors accounted for 97% of relative model probability (*w_i_*). Some equally parsimonious models included size measurements (total body length, weight) as well, but these did not significantly improve the model (Table 1). In the most parsimonious GLM including sex, species, and their interaction, males of *A. pseudonietana* were estimated to have similar *ΔΦ_y_* values to females (*t* = 29.327, *P* = 0.11, GLM), while males of *D. carolina* and *S. equale* had values that were significantly coarser (0.23° and 0.28°, respectively, all *P* < 0.05, GLM).

**Table 1:** Summary of predictor combinations explaining interommatidial angle perpendicular to the horizon (*ΔΦ_y_*)

Model	ΔAIC	*l_i_*	*w_i_*
*ΔΦ_y_* ∼Sex * Species	**0**	**1.00**	**0.42**
*ΔΦ_y_* ∼ Sex * Species + Weight	**1.1**	**0.58**	**0.24**
*ΔΦ_y_* ∼ Sex * Species + Length	**1.4**	**0.50**	**0.21**
*ΔΦ_y_* ∼ Sex + Species	4.6	0.10	0.04
*ΔΦ_y_* ∼ Sex + Species + Length	5.2	0.07	0.03
*ΔΦ_y_* ∼ Sex + Species + Weight	5.3	0.07	0.03
*ΔΦ_y_* ∼ Sex + Weight	6.9	0.03	0.01
*ΔΦ_y_* ∼ Sex	7.2	0.03	0.01
*ΔΦ_y_* ∼ Sex + Length	7.9	0.02	0.01
*ΔΦ_y_* ∼ Weight	17.4	0.00	0.00
*ΔΦ_y_* ∼ Length	28.3	0.00	0.00
*ΔΦ_y_* ∼ Species	35.5	0.00	0.00

ΔAIC values were calculated relative to the best fit model. The models in bold are equally parsimonious (within 2 ΔAIC). *l_i_* = model’s relative likelihood, *w_i_* = model probability.

#### Interommatidial angle parallel to the horizon (Δ*Φ_x_*)

In contrast to *ΔΦ_y_*, *ΔΦ_x_* showed no sexual dimorphism nor species-specific differences. *ΔΦ_x_* was not normally distributed (*P* < 0.01, *W* = 0.95755, Shapiro–Wilk normality test), and therefore a natural log transformation was used to restore normality. Neither sex (*P* = 0.18, df = 1, *F* = 1.86, two-way ANOVA), species (*P* = 0.07, df = 2, *F* = 2.73, two-way ANOVA), nor their interactions (*P* = 0.99, df = 2, *F* = 0.013, two-way ANOVA) were significant predictors of the natural log of *ΔΦ_x_* (Fig. 3B).

#### Dimorphism in the morphological determinants of Δ*Φ*

**Fig. 4:**
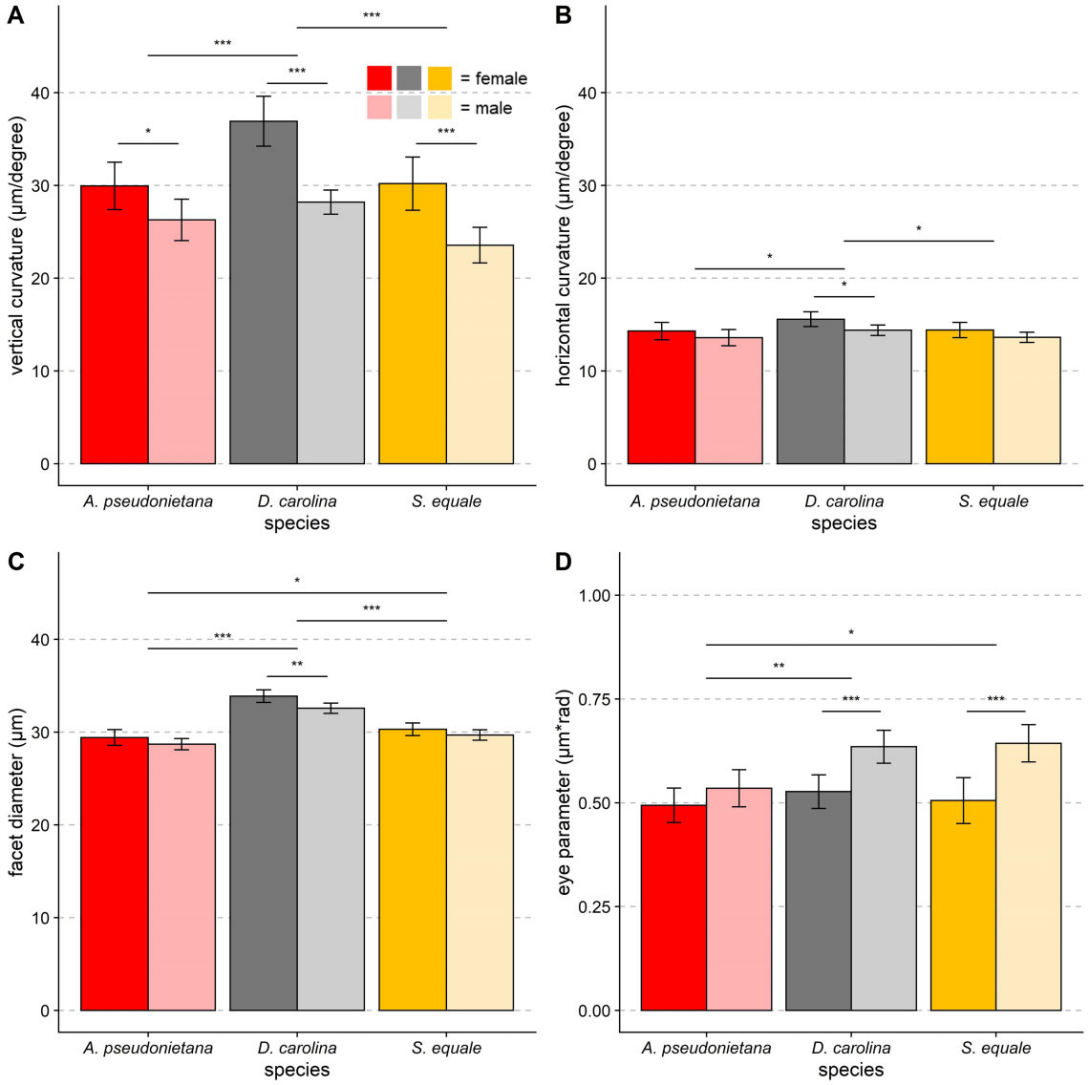
Morphological determinants of *ΔΦ* in band-winged grasshoppers. Eye curvature is the biggest morphological driver of the sexually dimorphic *ΔΦ* in these three species of band-winged grasshoppers. **A**) Biological females had ∼18% flatter vertical eye curvature in the region of the eye with the smallest *ΔΦ_s_* (*P* < 0.001, df = 1, *F* = 49.6, two-way ANOVA). **B**) Although significant, the differences in horizontal curvature were not as pronounced, as female curvature was only ∼6.5% flatter than males (*P* < 0.01, df = 1, *F* = 8.65, two-way ANOVA). **C**) Female facet diameters were slightly larger (∼2.8%) than male values (*P* < 0.01, df = 1, *F* = 1.52, two-way ANOVA). **D**) The observed eye parameter values are typical of diurnal insects and varied significantly between both sexes (*P* < 0.001, df = 1, *F* = 29.15, two-way ANOVA) and species (*P* < 0.001, df = 2, *F* = 2.92, two-way ANOVA). **A–D**) Sample sizes (from left to right) in each panel are 17, 18, 16, 16, 15, and 15 individuals, respectively. Error bars indicate 95% CI, significance symbols are based on *post hoc* analyses (Student’s *t*-test for sex differences, Tukey’s HSD for species differences).

At the morphological level, compound eyes can achieve finer *ΔΦ* if they have either 1) a flatter curvature or 2) smaller facets. In female band-winged grasshoppers, changes in eye curvature—and not facet size—contribute to finer *ΔΦ_y_* (Fig. 4). Vertical eye curvature was not normally distributed (*P* < 0.01, *W* = 0.95, Shapiro–Wilk normality test) because of the presence of one female *S. equale* outlier with a particularly flat eye (perpendicular eye curvature = 51 µm/degree). Removal of the outlier restored normality (*P* = 0.18, *W* = 0.98, Shapiro–Wilk normality test). Even with this especially flat eye removed, females still have a vertical curvature that is ∼18% flatter than their male counterparts (*P* < 0.001, df = 1, *F* = 49.56, two-way ANOVA, Fig. 4A). Additionally, there are species-specific differences in vertical eye curvature (*P* < 0.001, df = 2, *F* = 14.58, two-way ANOVA) with *post hoc* testing revealing that the larger *D. carolina* has a flatter vertical eye curvature than either of the two other species (*P* < 0.001 in both cases, Tukey’s HSD). The interactions between sex and species on vertical eye curvature were trending toward significance (*P* = 0.06, df = 2, *F* = 2.87), suggesting that the dimorphism in vertical curvature may vary between species.

Similarly to *ΔΦ_x_* and *ΔΦ_y_*, differences in eye curvature between the sexes were less pronounced in the horizontal axis than in the vertical axis (Fig. 4B). Both sex (*P* < 0.01, df = 1, *F* = 8.65, two-way ANOVA) and species (*P* < 0.01, df = 2, *F* = 4.85) significantly affected horizontal eye curvature. However, female horizontal curvature was only ∼6.5% flatter than males. There was no significant effect of the interaction between sex and species on horizontal eye curvature (*P* = 0.78, df = 2, *F* = 0.252).

Females had slightly larger *D* values than males (∼0.9 µm larger or 2.8%, Fig. 4C) across all three species measured (*P* < 0.01, df = 1, *F* = 11.47, two-way ANOVA). Additionally, all three species differed significantly in facet size (*P* < 0.001, df = 2, *F* = 96.06, two-way ANOVA) with the larger *D. carolina* having the largest facets (all *P* < 0.05, Tukey’s HSD). There was no significant effect of the interaction between sex and species *D* (*P* = 0.52, df = 2, *F* = 0.651, two-way ANOVA). The combination of changes in *ΔΦ* and facet size led to eye parameters that are of typical values for diurnal insects (Fig. 4D) but that varied significantly between both sex (*P* < 0.001, df = 1, *F* = 29.15, two-way ANOVA) and species (*P* < 0.001, df = 2, *F* = 2.92, two-way ANOVA). The interactions between sex and species on the eye parameter were trending toward significance (*P* = 0.059, df = 2, *F* = 2.918).

### Eye size and facet count variation in *D. carolina*

**Fig. 5:**
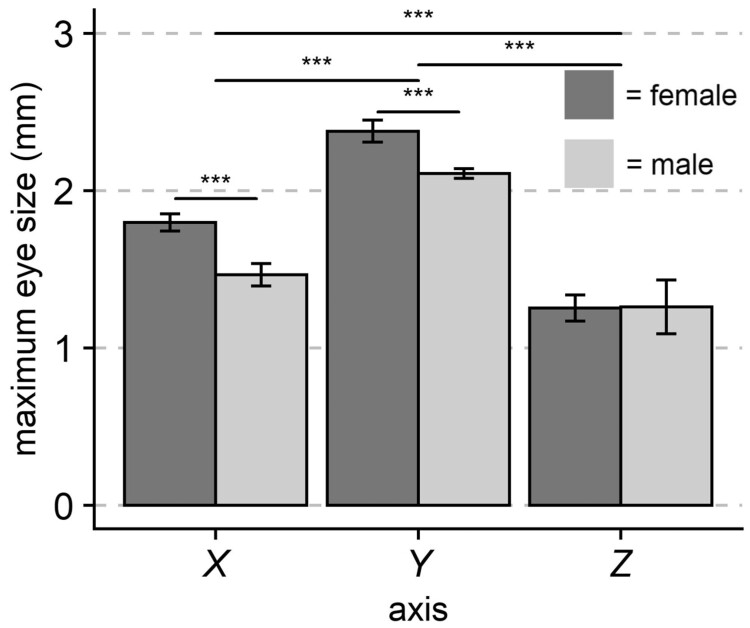
Eye size in the band-winged grasshopper *D. carolina*. Eye size is both asymmetrical and varies between the sexes. Females have larger maximum eye lengths in the *X*- and *Y*-axis, but not in exposed eye depth (*Z*; *P* < 0.001, df = 1, *F* = 28.90, two-way ANOVA). Exposed eye depth (*Z*) only measures exposed eye surface and may be an underestimate of total size. Sample sizes are 23 females and 25 males. Error bars indicate 95% CI, significance symbols are based on *post hoc* analyses (Student’s *t*-test for within axis differences, Tukey’s HSD for between axis differences).

Further investigation of the *ΔΦ_y_* dimorphism in the species that showed the greatest sexual size dimorphism (*D. carolina*) found that females typically have larger eyes than males, but not in every axis (Fig. 5). Compared with males, females had significantly larger maximum eye lengths in the horizontal (*X*; ∼13% increase) and vertical (*Y*, ∼23% increase) axes, but did not vary in exposed eye depth (*Z*; *P* < 0.001, df = 1, *F* = 28.90, two-way ANOVA; sex * interaction *P* < 0.001, df = 2, *F* = 7.98). Additionally, both male and female eyes varied significantly in diameter between the axes (*P* < 0.001, df = 2, *F* = 240.73).

As expected, based on differences in eye size and *D*, facet counts of a subset of representative individuals suggest that the female eyes have more facets than males (Table 2). Although there was variability in the number of facets seen in each sex, representative females (average = 5565 facets, *n* = 3) had eyes with ∼19% more facets than males (4679 facets, *n* = 3).

**Table 2:** Whole eye facet counts in representative *D. carolina* individuals

Eye size percentile	Female	Male
∼25th	4901 facets	4571 facets
∼50th	5514 facets	4453 facets
∼75th	6280 facets	5013 facets

## Discussion

Most band-winged grasshoppers have non-spherical eyes featuring an elongated vertical axis that gives them a kidney-bean like shape. Similar to other invertebrates with non-spherical eyes (Kelber and Somanathan 2019; Bagheri et al. 2020) this leads to better VA in the axis of elongation: in band-winged grasshoppers estimated interommatidial angle in the vertical axis (*ΔΦ_y_*) is around half the value of those as that in the horizontal axis (*ΔΦ_x_*; Fig. 3). *ΔΦ_y_* appears to be enhanced by a particularly flat vertical region near the center of the eye. Although no studies have previously examined *ΔΦ_s_* in North American species of band-winged grasshoppers, our values (mean female species *ΔΦ_y_* = 0.94°–1.00°, *ΔΦ_x_* = 2.09°–2.21°) are similar to what has been measured in females of two European species via the pseudopupil methodology (*Locusta migratoria ΔΦ_y_* = 1.09°, *ΔΦ_x_* = 2.4° [Burtt and Catton, 1954]; *Schistocerca gregaria* minimum *ΔΦ* = 0.95 [Krapp and Gabbiani, 2005]), and our eye parameter values are typical of other diurnal insects (Land, 1997). Our estimated interommatidial angles would correspond to VA values of VA_*y*_ = 1.9°–2°, and VA_*x*_ = 4.2°–4.4° in the flattest region of the eye.

The ∼two-fold difference between *ΔΦ_y_* and *ΔΦ_x_* seen in this study is not uncommon for arthropod eyes, yet its function and/or selective benefit is poorly understood. Many species of bees have teardrop like eyes that result in finer vision in the vertical axis (reviewed in Kelber and Somanathan 2019) that is of similar magnitude to the difference observed here (e.g., Shaw 1969; Dyer et al. 2016). That bees and band-winged grasshoppers are both diurnal fliers suggest that this arrangement could potentially be beneficial when considering the effects of flight and motion blur. Alternatively, the horizontal streak in fiddler crabs also shows a similar improvement to vertical versus horizontal vision (Land and Layne 1995; Zeil and Al-Mutairi 1996; Zeil and Hemmi 2006). This zone may assist in predator avoidance in a relatively flat environment (Zeil and Hemmi 2006), drawing parallels to predator avoidance behavior in band-winged grasshoppers. Future studies that more carefully examine the topography of vision or the behavior of the grasshoppers can further elucidate why eye size, curvature, and *ΔΦ* are asymmetric between the axes.

Within the more acute vertical axis, *ΔΦ_y_* is sexually dimorphic, resulting in female grasshoppers—the larger of the two sexes—having finer *ΔΦ_y_* values in two of the three species examined (Fig. 3A). Similar to other grasshopper species (Otte 1984; Hochkirch and Gröning 2008), female band-winged grasshoppers were substantially larger than males in this study (Fig. 2). Similarly, *ΔΦ_y_*—but not *ΔΦ_x_*—was sexually dimorphic, with females having ∼19% smaller values than males in the flattest region of the eye (Fig. 3A and B). This *ΔΦ_y_* dimorphism is similar in magnitude to the classic “love spot” seen in male flies (although with only minor accompanying sensitivity specializations [Land and Eckert, 1985]). However it is in the opposite direction: females have finer vision rather than males. This suggests that much like between species differences (Kiltie 2000; Land and Nilsson 2012; Veilleux and Kirk 2014; Caves et al. 2017, 2018), size can lead to finer vision within a species. Notably, within either sex there was no further effect of size on *ΔΦ_y_* (Fig. 3C and Supplementary Fig. S2). Thus, in some cases the positive relationship between acuity and size could be constrained to individuals with consistently different developmental processes such as sex and/or morph differences.

In *D. carolina* and *S. equale*, the dimorphism in *ΔΦ_y_* was due to a flatter eye surface and not a reduction in *D* (Fig. 4). Although both changes can lead to finer acuity, smaller *D* values are not typically seen in insects because it may also reduce overall sensitivity (Kirschfeld 1976; Land 1997). The acute zones of many insects require both fine VA and high sensitivity (Warrant 2016), and therefore often feature flat regions with large facets. In band-winged grasshoppers, females either had similar or slightly larger facets than males. However, their eye’s flatter curvature led to a substantial *ΔΦ_y_* dimorphism. As a result, female band-winged grasshoppers have finer estimated *ΔΦ_y_* without sacrificing sensitivity by decreasing *D* (Fig. 4).

Further study in *D. carolina* suggests that the sexual size dimorphism corresponds with both an eye size dimorphism (Fig. 5) and a facet number dimorphism (Table 1). Altogether, this suggests that female *D. carolina* improve *ΔΦ_y_* by having larger eyes with more facets. Surprisingly, we also found that females had larger eyes in the horizontal axis, despite showing no improvement in *ΔΦ_x_* compared with males. This increase in *X* eye size suggests that female *D. Carolina* may improve VA, sensitivity, and/or field of view in other regions of their eye that were not measured within this study. Future studies should further explore the regional variation within female and male eyes to elucidate how visual performance changes outside of the central region of the eye.

Although the magnitude of the sex dimorphism varies by both parameter and species (Table 3), in general *D. carolina* and *S. equale* had visual dimorphisms in the vertical axis that were of equal magnitude or greater than the corresponding body length dimorphism. This is surprising as eyes are generally metabolically expensive and rarely scale isometrically with body size (Kiltie 2000; Jander and Jander 2002; Veilleux and Kirk 2014; Caves et al. 2017). An isometric to positive allometric scaling of vertical axis parameters suggests that improved vision in female *D. carolina* and *S. equale* may be under selection and not solely a result of increasing body size. Notably, this is not the case for every visual parameter (e.g., *D*, horizontal curvature) nor for every species; although *A. pseudonietana* has a size dimorphism that is similar to other species, it showed no statistically significant dimorphism in any of the parameters expected to improve vision (*ΔΦ_y_*, *ΔΦ_x_*, facet diameter, Figs. 3 and 4 and Table 3). Further behavioral and/or morphological examinations of this species could determine why the relationship between sex and vision is different than those in the other species examined.

**Table 3:** Sexual dimorphism magnitude across parameters (female value/male value)

	Species		
Parameter	*A. pseudonietana*	*D. carolina*	*S. equale*
Length	**1.15**	**1.21**	**1.14**
1/*ΔΦ_y_*	1.08	**1.25**	**1.28**
1/*ΔΦ_x_*	1.02	1.03	1.03
*D*	1.02	**1.04**	1.02
*Y* curvature	**1.14**	**1.31**	**1.28**
*X* curvature	1.05	**1.08**	1.06
Eye size (*X*)	–	**1.23**	–
Eye size (*Y*)	–	**1.13**	–
Eye size (Z)	–	0.99	–

Significant differences in bold, see text for details.

It is unknown what—if any—selective advantage female *D. carolina* or *S. equale* may gain from having larger eyes with both finer vision and larger facets. One possibility is that females could benefit by more accurately interpreting visual signals. Although mating systems and behaviors are variable across band-winged grasshoppers, many species use a variety of potential visual signals including those involving either their colorful hindwings or leg movements (Willey and Willey 1969; Otte 1970, 1984; Kerr 1974). Females may therefore benefit by being better able to perceive and interpret these potential signals of species identity and mate quality, and the smaller *ΔΦ_y_* values could be especially useful for detecting leg motion signals with a strong vertical component. Additionally, females could use their enhanced vision to initiate anti-predator defenses at a greater distance. Band-winged grasshoppers have a suite of defensive behaviors in response to approaching predators (Santer et al. 2012; Collier and Hodgson 2017). Initiating these defenses at a greater distance could be especially beneficial to females because they may take a longer time than males to reach sexual maturity even once in their final adult instar (Pfadt 1994). Alternatively, the visual dimorphism could be a byproduct of increased body size and serve no beneficial function. Notably, we only measured morphological correlates of vision in this study and did not examine any accompanying increases in brain power and/or behavioral outcomes. However, in compound eyes differences in morphological parameters are often associated with accompanying changes in physiology and neurobiology (Land 1997), especially when considering the metabolic costs of eyes (Laughlin 2001; Niven and Laughlin 2008) and scaling observed in this study (Table 3). Future studies utilizing the natural variation in band-winged size dimorphism, mating systems, and development could elucidate how these factors contribute to the visual dimorphisms seen in this study. Additionally, a more robust angular mapping of band-winged grasshopper eyes could better help understand what stimuli might fall within the regions of dimorphic vision.

**Fig. 6:**
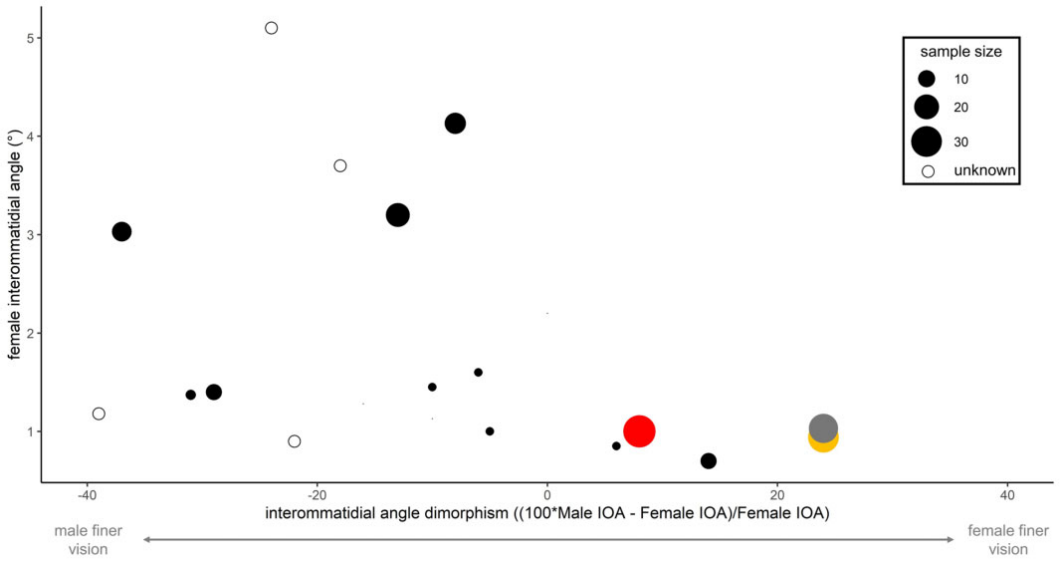
In insects with moderate spatial vision (*ΔΦ*  <  5°), studies that report Δ*Φ* separately for each sex have typically 1) shown males with smaller *ΔΦ_s_* than females and 2) been based on relatively small sample sizes. Our study (colored circles) is one of the first to suggest a female-biased *ΔΦ* dimorphism in insects with moderate or better spatial vision (*ΔΦ* < 5°). Red = *A. pseudonietana*, yellow = *S. equale*, gray = *D. carolina*. Data and references can be found in Supplementary Table S1.

Surprisingly, our results are one of the first studies to show females with finer vision than males (Fig. 6). In insects, females of the tiny parasitic wasp *Trichogramma evanescens* have larger eyes with ∼23% finer vision than males (Fischer et al. 2011). However, because of their small size, female VA (∼20°) is much coarser than what was measured in this study and likely limits their visually guided behaviors (Nilsson 2009, 2013). In mammals, Seymoure and Juraska (1997) found that female rats behaviorally outperform males for coarse stimuli but that this sex difference disappeared at finer stimuli. And in fish, Corral-Lόpez et al. (2017) found that female artificially selected guppies have finer VA than males. However, unlike what we report in this study, the difference disappears when controlling for body size.

We believe that the documentation of females with finer vision than males is lacking—not because it is a rare phenomenon—but rather little research has been conducted on the topic. Aside from the well documented love-spot in species of flying insects, few VA studies have examined differences between the sexes and studies often do not report the sexes of the individuals measured. Instead, most studies have prioritized either an ecological approach (sampling only a few individuals because they sample many species; e.g., Pettigrew et al. 1988; Collin and Partridge 1996; Lisney et al. 2012) or a retinal topography approach (sampling only a few individuals because of the extensive work it takes in each individual; e.g., Coimbra et al. 2013; Landgren et al. 2014) making extensive comparisons between the sexes difficult. More recent studies in insects have found variation in eye-scaling within insect species but have so far focused on the morphs of eusocial insects (Perl and Niven 2016a, 2016b; Taylor et al. 2019). Because eye size is a major factor influencing visual performance (Kirschfeld 1976; Land and Nilsson 2012; Cronin et al. 2014), body size increases that lead to eye size increases could be utilized for VA improvements (Corral-López et al. 2017). Based on the prevalence of sexual size dimorphisms throughout many animal groups (Parker 1992; Lislevand et al. 2007; Teder 2014; Kuntner and Coddington 2020), VA differences between the sexes may be an under-documented—yet not uncommon—phenomenon that warrants further consideration and exploration.

## Supplementary Material

obab008_Supplementary_Data
