# Supplementary material for: A Sexual Dimorphism in the Spatial Vision of North American Band-Winged Grasshoppers
Source: Integr Org Biol. 2021 Apr 7;3(1):obab008. doi: 10.1093/iob/obab008 (PMC8358991; doi:10.1093/iob/obab008)
Supplement: obab008_Supplementary_Data [file obab008_supplementary_data.zip › Abstract (Spanish).docx]

**Resumen**

La agudeza visual (AV)--- una medida de la finura o la dificultad visual--- puede variar dentro de una especie, incluso entre los sexos biológicos. Aunque numerosos estudios han encontrado machos con una AV más fina que las hembras, relativamente pocos han demostrado lo contrario, hembras con visión más fina. Esto es sorprendente porque nuestra comprensión de diferencias entre especies en AV sugiere que las hembras pueden tener una visión más fina que los machos si 1) son más grandes que los machos, o 2) necesitan una visión más fina para detectar y/o discriminar entre los machos. Aquí, estimamos el ángulo interommatidial (∆Φ, una medida anatómica de AV) en tres especies de saltamontes de ala de banda en las que las hembras son el sexo más grande y probablemente interpretan señales visuales (Arphia pseudonietana, Dissosteira carolina, y Spharagemon equale; total n = 98). Usando un método de estimación de radio de curvatura, encontramos que las hembras tienen un estimado ∆Φ ~19% más fino que los machos en la región y eje más agudos del ojo, pero que este dimorfismo varía entre especies. Exploraciones visuales adicionales de la especie que muestra el mayor dimorfismo del tamaño corporal (D. carolina) sugieren que este dimorfismo de ∆Φ debe a que las hembras tienen ojos más grandes con más omatidios. En contraste con muchos insectos voladores diurnos donde los machos tienen una visión más fina para adquirir parejas, nuestro estudio es uno de los primeros en demostrar un dimorfismo sesgado por las hembras en la agudeza. Dado 1) el número de especies en las que las hembras son más grandes que los machos, y 2) la variabilidad de los comportamientos de apareamiento entre taxones, nuestros resultados sugieren que las diferencies de AV entre los sexos pueden ser más comunes de lo que se aprecia actualmente
